# The Role of Beneficial Microbiota in COVID-19: Insights from Key Bacterial Genera

**DOI:** 10.3390/microorganisms13051029

**Published:** 2025-04-29

**Authors:** Pabulo Henrique Rampelotto, Clarissa Reginato Taufer, Juliana da Silva

**Affiliations:** 1 Bioinformatics and Biostatistics Core Facility, Instituto de Ciências Básicas da Saúde, Universidade Federal do Rio Grande do Sul, Porto Alegre 91501-970, Brazil; 2 Graduate Program in Genetics and Molecular Biology, Universidade Federal do Rio Grande do Sul, Porto Alegre 91501-970, Brazil; 3 Graduate Program in Health and Human Development, Universidade La Salle, Canoas 92010-000, Brazil

**Keywords:** probiotics, *Akkermansia*, *Bacteroides*, *Faecalibacterium*, dysbiosis, immune modulation, viral infections, microbiota-targeted therapy

## Abstract

The COVID-19 pandemic has highlighted the need for a comprehensive understanding of the factors influencing disease severity and progression. Emerging research indicates that the human microbiota, particularly beneficial bacteria, significantly impacts immune responses and health outcomes in COVID-19 patients. While existing studies provide general insights into the relationship between the microbiota and probiotics with COVID-19, they often lack a detailed exploration of how specific bacterial taxa might be used as adjunctive treatments. This review aims to address this gap by focusing on ten key genera of beneficial bacteria, discussing their roles in COVID-19 and evaluating their potential as probiotics for prevention and treatment. The review covers the impact of these microbes on human health, their population alterations in COVID-19 patients, and their interactions with other viral infections. Among these microbes, several exhibit distinct patterns of abundance in COVID-19 patients, influencing disease outcomes and highlighting their potential roles in infection dynamics. In COVID-19 patients, populations of *Akkermansia*, *Ruminococcus*, and *Roseburia* are consistently reduced, while those of *Faecalibacterium* show a significant decline in more severe cases. *Bacteroides* presents varying effects depending on the species involved. Alterations in the abundance of *Blautia* and Lachnospiraceae are associated with increased inflammation and disease severity. Likewise, the depletion of *Lachnospira* and *Coprococcus* populations, both linked to anti-inflammatory effects, may exacerbate symptom severity. Oscillospira, though less studied, is connected to overall health and could have implications for viral infections. This review synthesizes the current understanding of these beneficial microbes to highlight the importance of maintaining a healthy microbiota to alleviate the impact of COVID-19 and contribute to the development of novel therapeutic strategies involving microbiota modulation.

## 1. Introduction

The human microbiota plays a significant role in maintaining health and regulating immune responses [1]. Beneficial bacteria within these microbial communities are essential for various physiological processes, such as digestion, vitamin synthesis, and defense against pathogens [2]. Maintaining the delicate balance of the microbiota is the key to homeostasis, as disruptions can contribute to metabolic disorders, inflammatory diseases, and infections [3,4].

The COVID-19 pandemic, caused by the novel coronavirus SARS-CoV-2, has brought unprecedented challenges to global health. While much attention has been focused on the direct impact of the virus on respiratory and systemic health, several studies demonstrate that the gut microbiota may also play a significant role in disease progression and severity. Alterations in the composition and function of the gut microbiota have been observed in COVID-19 patients, indicating a potential link between microbial dysbiosis and the clinical outcomes of the disease [5,6]. Clinical studies indicate that COVID-19 patients exhibit significant alterations in their gut microbiota, with a reduction in levels of beneficial bacteria and an increase in levels of opportunistic pathogens [6]. This dysbiosis is associated with disease severity and inflammatory markers, highlighting the importance of maintaining a healthy microbiota [7].

Probiotics, live microorganisms that provide health benefits to the host when administered in sufficient amounts, have been widely studied for their potential to modulate immune responses and support gut health during infections, including COVID-19. Probiotic supplementation could therefore be a valuable adjunctive therapy in managing COVID-19, helping to restore microbial balance and improve clinical outcomes (Mak et al., 2020) [8]. Numerous studies demonstrate that probiotics can enhance the gut barrier function, modulate systemic inflammation, and influence the immune system’s response to viral infections [9,10,11].

In COVID-19, probiotics may offer several potential benefits. They can help restore the balance of the gut microbiota disrupted by the viral infection and associated treatments, such as antibiotics [8]. Probiotics like *Bifidobacterium* and *Lactobacillus* enhance the production of antiviral cytokines and reduce the severity of respiratory infections [12,13]; however, it remains unclear to what extent diet and medications might influence the efficacy of probiotics in restoring the gut microbiota balance during COVID-19 [14]. In addition, some probiotic strains reduce the duration and severity of symptoms in viral infections [15], suggesting a potential role in alleviating COVID-19 symptoms. In previous reviews, we detailed the involvement of *Bifidobacterium* and *Lactobacillus* in COVID-19 [16,17,18]. However, the roles of other well-known probiotics have yet to be thoroughly reviewed, as the literature on these genera is scattered, making it difficult to achieve a clear understanding.

This review aims to explore the relationship between ten key genera of beneficial or probiotic bacteria and COVID-19. Each section discusses the influence of these genera on human health, their population alterations in COVID-19 patients, and their interactions with other viral infections, which can contribute to understanding the relationship between these genera and viral infections. This knowledge could facilitate the development of innovative therapeutic strategies aimed at restoring microbial balance and enhancing host defenses against SARS-CoV-2 and other viral pathogens.

## 2. Key Bacterial Genera

### 2.1. Akkermansia

*Akkermansia*, particularly the species *Akkermansia muciniphila*, is a beneficial bacterium that plays a significant role in maintaining gut barrier integrity and modulating immune responses. This mucin-degrading bacterium resides in the mucus layer of the gut, contributing to the maintenance of the gut lining and promoting overall mucosal health [19]. *Akkermansia* abundance is inversely associated with various metabolic disorders, including obesity, type 2 diabetes, and metabolic syndrome [20,21]. Its presence is linked to reduced inflammation and improved insulin sensitivity, indicating its potential as a therapeutic target for metabolic diseases [22].

In COVID-19, an increase in *Akkermansia* populations has been observed in both infected patients and those who have recovered [23,24,25,26,27]. This increase correlates with worsening disease severity and higher rates of SARS-CoV-2 viral replication. Specifically, patients exhibiting lower viral loads (Ct > 31) showed decreased *Akkermansia* levels compared to those with higher replication rates (Ct ≤ 31) [28]. These findings suggest a relationship between disease severity, viral load, and altered *Akkermansia* abundance, indicating that dysbiosis may persist even after recovery from COVID-19. In addition, the enrichment of *Akkermansia muciniphila* populations has been positively associated with levels of pro-inflammatory cytokines such as IL-1β, IL-6, and CXCL8 [7], which could imply potential protective effects during COVID-19. However, *Akkermansia* abundance was reduced in the oral microbiota of post-COVID-19 patients (1–8 months after infection) and in those who received antibiotics during treatment [29,30], as well as in children [31,32]. Furthermore, this genus was enriched in patients with other respiratory diseases compared to COVID-19 patients in intensive care units, emphasizing the complex relationship between *Akkermansia* and respiratory illnesses [33].

The involvement of *Akkermansia* in other viral infections has also been investigated. Its ability to strengthen the gut barrier and modulate immune responses suggests a protective role in viral infections affecting the gastrointestinal tract. An increase in *Akkermansia* abundance was reported in children living with HIV undergoing antiretroviral treatment, potentially linked to the beneficial effects of this treatment on inflammation [34]. In addition, *Akkermansia muciniphila* may protect hosts from Severe Fever with Thrombocytopenia Syndrome (SFTS) by attenuating systemic inflammation through the production of the β-carboline alkaloid harmaline, which suppresses NF-κB-mediated inflammation [35].

In animal models, an increase in *Akkermansia* abundance was observed following influenza A virus infection, particularly on day 7 post-infection [36]. Similarly, in Severe Fever with Thrombocytopenia Syndrome virus-infected mice, those that survived showed a significant increase in *Akkermansia muciniphila* abundance three days post-infection [35]. By contrast, H3N2-infected mice exhibited lower levels of *Akkermansia* [37]. The increase in *Akkermansia muciniphila* abundance was associated with rapidly spreading viral infections, and CD8 T cells were shown to modulate several dysbiotic taxa after lymphocytic choriomeningitis virus (LCMV) infection in mice, resulting in significant anorexic behavior [38]. *Akkermansia muciniphila* not only proliferated following LCMV infection but also during fasting conditions. Notably, experimental enrichment of *Akkermansia muciniphila* populations after LCMV infection suppressed the expression of Granzyme B and T-BET in virus-specific CD8 T cells, suggesting that natural enrichment may help mitigate initial immune responses.

This variability highlights the complexity of *Akkermansia*’s role in viral infections, with evidence suggesting that its increased abundance is associated with fast-spreading viruses and that it may have the potential to modulate immune responses and maintenance of the gut lining, which could be beneficial in mitigating the effects of viral infections, including in cases of COVID-19.

### 2.2. Bacteroides

*Bacteroides* is a dominant genus in the human gut microbiota, playing a crucial role in the digestion of complex carbohydrates and the production of short-chain fatty acids (SCFAs), which are essential for gut health [39]. As commensal bacteria, *Bacteroides* species contribute to the maintenance of gut homeostasis; however, imbalances in their populations have been associated with various diseases, including inflammatory bowel disease (IBD) and obesity [40,41]. The impact of *Bacteroides* on inflammation varies between species: some protect against excessive inflammation, while others can exacerbate inflammatory responses, highlighting the importance of species composition in shaping clinical outcomes. Additionally, certain species may act as opportunistic pathogens, causing infections outside the gut, such as bacteremia and abscesses [42,43].

In COVID-19, several studies have reported significant alterations in *Bacteroides* populations. Al Bataineh et al. (2020) observed a reduction in *Bacteroides* abundance among COVID-19 patients compared to healthy controls, suggesting that SARS-CoV-2 may disrupt the gut microbiota, leading to a loss of beneficial *Bacteroides* species [44]. On the other hand, Reinold et al. (2021) and Mizutani et al. (2020) found an increase in the relative abundance of *Bacteroides* in patients positive for SARS-CoV-2 [45,46]. Bucci et al. (2023) further identified *Bacteroides* as one of the 10 key variables predicting COVID-19 severity [47], and Galperine et al. (2023) recognized it as a signature genus in COVID-19 patients with diarrhea [48]. In addition, an increase in the abundance of the opportunistic pathogen *Bacteroides nordii* was observed in the feces of COVID-19 patients [6]. Worth noting, *Bacteroides dorei*, *Bacteroides thetaiotaomicron*, *Bacteroides massiliensis*, and *Bacteroides ovatus* populations were inversely correlated with SARS-CoV-2 load in fecal samples [6], with *Bacteroides dorei* downregulating ACE2 expression in the murine colon, a key receptor for SARS-CoV-2 entry [49]. These findings demonstrate the complexity of microbiota alterations in COVID-19 and suggest that the impact on *Bacteroides* may vary depending on the study population, disease severity, and specific species involved.

*Bacteroides* has also been studied in the context of other viral infections. The oropharyngeal microbiota of patients with influenza A virus had a reduced abundance of *Bacteroides* [50]. In addition, *Bacteroides* species have been implicated in modulating immune responses during viral infections. *Bacteroides fragilis*, for example, produces polysaccharide A, which can enhance the immune response and protect against viral encephalitis in mice [51]. Polysaccharide A also induces the expression of IFN-β, which regulates natural resistance to viral infections, including influenza virus A, in murine models and in vitro analyses [52] and protects against intestinal inflammation by inducing IL-10-producing CD4+ regulatory T cells [53]. In mice infected with H3N2, a lower abundance of *Bacteroides* was observed, along with reduced levels of acetate in the intestine and serum [37]. *Bacteroides* produce acetate, and this SCFA could enhance the host’s antiviral response [54], reduce inflammatory reactions, and prevent lung injuries [55]. Furthermore, pretreatment with acetate in H3N2-infected mice was able to partially restore the airway epithelial barrier function and reduce the levels of inflammatory cytokines TNF-α, IL-6, and IL-1β in the lungs [37]. Changes in *Bacteroides* populations have also been observed in HIV-infected individuals, with a shift toward a more inflammatory gut microbiota [56]. These findings suggest the role of *Bacteroides* in both maintaining immune homeostasis and responding to viral infections. Maintaining a healthy population of *Bacteroides* may be beneficial in mitigating the effects of viral infections and could be effective in reducing disease severity, including in cases of COVID-19.

### 2.3. Blautia

*Blautia* are known for their ability to produce SCFAs, particularly acetate and butyrate, which are beneficial for gut health and have anti-inflammatory [57]. *Blautia* species are associated with a healthy gut microbiota and have been inversely correlated with various diseases. Reduced levels of *Blautia* have been observed in patients with IBD, obesity, type 2 diabetes, and some types of cancer [57,58]. Consequently, a reduction in *Blautia* abundance has been implicated in increased systemic inflammation and disease severity.

In COVID-19, several studies have documented alterations in the abundance of *Blautia*. Al Bataineh et al. (2020) reported an increase in the abundance of this genus in COVID-19 patients [44], while Maeda et al. (2022) and Gaibani et al. (2021) identified *Blautia* as one of the characteristic bacteria in healthy controls [23,59]. Gaibani et al. (2021) also observed that *Blautia* was associated with COVID-19 patients who did not require ICU admission or develop bloodstream infections, suggesting a potential protective role for this taxon [23]. Wu et al. (2021) identified reduced *Blautia* abundance in the gut microbiota of COVID-19 patients and proposed it as a potential biomarker due to its decreased levels [60]. Similarly, Schult et al. (2022) reported a reduced abundance of *Blautia* in severe and fatal cases, indicating that lower levels of this beneficial bacterium may be linked to worse disease outcomes [61]. In nasopharyngeal samples, Romani et al. (2024) observed a reduction in *Blautia* populations in children with COVID-19, with the genus being negatively associated with the disease [32]. Collectively, these findings suggest that *Blautia* may play a protective role in COVID-19, and its reduced abundance could be associated with disease progression and severity.

In other viral infections, a reduction in the abundance of *Blautia* has been consistently observed and linked to reduced inflammation. Gu et al. (2020) found significant depletion of this genus in the gut microbiota of influenza A patients [5]. In children with rotavirus, combining conventional therapy with zinc supplementation showed a negative correlation between *Blautia* abundance and levels of inflammatory markers like IL-6, TNF-α, PCR, and D-lactate [62]. Animal studies demonstrated that *Blautia faecis*, isolated from post-influenza mice, reduced IL-8 production induced by TNF-α in intestinal epithelial cells [63]. In HIV-positive patients, Vesterbacka et al. (2017) reported reduced *Blautia* levels, with higher levels found in antiretroviral therapy-naïve patients compared to elite controllers, indicating a positive association with immune activation [64]. Mutlu et al. (2024) observed increased *Blautia* abundance in control groups [65], while Vázquez-Castellanos et al. (2015) identified it as a biomarker for these groups [66]. In co-infected HIV and hepatitis C virus (HCV) patients, direct-acting antiviral treatment increased *Blautia* abundance, suggesting microbiota restoration post-treatment [67].

### 2.4. Coprococcus

*Coprococcus* is also an SCFA-producing genus of Lachnospiraceae. The genus has been associated with various diseases, primarily due to its role in gut health and overall microbiome balance. Decreased levels of *Coprococcus* have been linked to obesity, with studies showing that its abundance is reduced in obese individuals, which indicates a potential role in metabolic regulation and energy homeostasis [68,69]. In addition, lower levels of *Coprococcus* have been observed in IBD, contributing to dysbiosis that exacerbates inflammation and gastrointestinal symptoms [70,71]. Furthermore, recent studies indicate an association between *Coprococcus* and neurodegenerative diseases, such as Parkinson’s disease, where gut microbiota imbalances may influence neuroinflammation and the gut–brain axis [72,73].

In COVID-19, several studies have reported alterations in *Coprococcus* populations. Albrich et al. (2022) found lower *Coprococcus* levels in high-risk COVID-19 groups compared to healthy controls [74], while Wu et al. (2021) identified its reduction as a potential biomarker for COVID-19 [60]. Gaibani et al. (2021) further noted that *Coprococcus* populations were enriched in COVID-19 patients who did not require ICU admission compared to both COVID-19 and non-COVID-19 patients in the ICU, suggesting a possible protective or modulatory role in the severity of the disease [23]. Similarly, Schult et al. (2022) observed a reduction in *Coprococcus* abundance in severe and fatal cases of COVID-19, implying that lower levels of this beneficial bacterium may be linked to worse outcomes [61]. In post-COVID-19 patients, Zhang et al. (2023a) reported a lower relative abundance of *Coprococcus* compared to healthy controls [75]. At the species level, Li et al. (2021) [76] reported a reduction in *Coprococcus catus* populations in COVID-19 patients compared to controls, with an inverse association observed between *Coprococcus catus* abundance and IL-8 levels in post-COVID-19 patients, suggesting that its reduction may contribute to increased inflammation that persists after recovery [77]. Altogether, these findings indicate that disruptions in *Coprococcus* populations may contribute to both the severity and the prolonged impact of COVID-19.

Although the role of *Coprococcus* in other viral infections has not been extensively studied, its known benefits in maintaining gut health and modulating immune responses suggest a potential protective role. The production of butyrate and propionate by *Coprococcus* is a key factor for enhancing gut barrier integrity and reducing systemic inflammation, both of which are essential during viral infections [78]. In HIV, dysbiosis and reduced *Coprococcus* levels have been linked to increased systemic inflammation and immune activation [79].

### 2.5. Faecalibacterium

*Faecalibacterium*, particularly *Faecalibacterium prausnitzii*, is a prominent and beneficial member of the human gut microbiota. It is a major producer of butyrate, an SCFA that serves as an energy source for colonic epithelial cells and exhibits anti-inflammatory properties [80]. *Faecalibacterium prausnitzii* abundance has been inversely associated with inflammatory conditions such as Crohn’s disease and ulcerative colitis, suggesting its protective role in gut health [81,82,83]. As one of the primary producers of butyrate, the reduction in *Faecalibacterium prausnitzii* populations, particularly in severe cases of COVID-19, suggests a breakdown of the intestinal barrier and an increase in systemic inflammation. Reduced levels of *Faecalibacterium* have also been observed in patients with obesity, type 2 diabetes, and IBD, further highlighting its importance in maintaining metabolic and gastrointestinal health [84,85,86].

In COVID-19, studies have shown varying effects on *Faecalibacterium* populations. While Al Bataineh et al. (2020) observed an increase in the abundance of this bacterium in COVID-19 patients compared to healthy controls, several other studies reported a decrease [44]. Bucci et al. (2023) identified *Faecalibacterium* as a biomarker for predicting disease severity [47], but Albrich et al. (2022) [74], Maeda et al. (2022) [59], Reinold et al. (2021) [45], Mizutani et al. (2020) [46], Schult et al. (2022) [61], Hazan et al. (2022) [87], and Yeoh et al. (2021) [7] all noted reduced levels in patients with severe or high-risk conditions. Specifically, the abundance of *Faecalibacterium prausnitzii* was inversely correlated with the severity of COVID-19, highlighting its potential protective role in severe COVID-19 cases [6]. These data suggest that lower levels of this beneficial bacterium may be linked to worse disease outcomes, while its presence could help fight infection. In addition, *Faecalibacterium prausnitzii* abundance was reduced in samples collected up to 30 days after disease resolution and for at least 6 months after recovery in severe patients, demonstrating the delay in restoring a healthy microbiota and its potential implications for post-COVID-19 health [7,59]. Tang et al. (2020) identified that *Faecalibacterium prausnitzii* abundance was negatively related to PRC in the critical COVID-19 group [88]. It is also important to highlight that the reduction in the diversity of *Faecalibacterium* observed in patients with bloodstream infections [89] was comparable to the significant decrease in diversity seen in severely ill mice intentionally infected with SARS-CoV-2 [90,91]. These findings indicate that *Faecalibacterium* levels may be disrupted in COVID-19, with potential implications for disease severity, progression, and inflammation.

Studies on other viral diseases have also reported a reduction in *Faecalibacterium* populations. In H1N1 patients, the abundance of *Faecalibacterium* was reduced compared to healthy controls [5], and in a Chinese cohort, *Faecalibacterium* abundance was negatively associated with the risk of H1N1 severity [92], indicating a protective role in the infection. *Faecalibacterium* abundance was also reduced in patients with H7N9, especially in those treated with antibiotics [93]. In addition, *Faecalibacterium prausnitzii* was linked to anti-inflammatory effects both in vitro and in vivo models of colitis [83]. *Faecalibacterium* populations were also consistently depleted among HIV+ individuals, which can contribute to inflammation and immune dysregulation, exacerbating the disease’s progression and affecting overall health [94].

### 2.6. Lachnospira

*Lachnospira* is also an SCFA-producing genus of Lachnospiraceae. The genus has been associated with various diseases, particularly due to its role in maintaining gut health and influencing systemic inflammation. Decreased levels of *Lachnospira* were linked to obesity and metabolic syndrome, with studies indicating that lower abundance of this genus correlates with increased body mass index and insulin resistance [95,96]. In IBD, reduced *Lachnospira* levels were observed, suggesting a potential role in gut barrier function and immune modulation, which may exacerbate inflammatory responses [70,71]. Furthermore, *Lachnospira* has been implicated in neurodegenerative conditions, including Alzheimer’s disease, where dysbiosis and altered gut microbiota composition may influence neuroinflammation and cognitive decline [97,98].

In COVID-19, some studies have reported changes in *Lachnospira* populations. Albrich et al. (2022) observed that *Lachnospira* levels were lower in high-risk COVID-19 groups compared to healthy controls [74]. Moreira-Rosário et al. (2021) also reported a significant reduction in *Lachnospira* populations in COVID-19 patients, particularly in those with severe disease [99]. Gaibani et al. (2021) identified the genus as one of the main discriminants of the microbiota in healthy controls [23]. Romani et al. (2022) identified the absence of *Lachnospira* in patients with moderate COVID-19 compared to patients with mild and asymptomatic cases, suggesting that the presence or absence of *Lachnospira* may be linked to the severity of the disease [31].

Some studies have also observed changes in *Lachnospira* abundance in other viral infections. In HIV, *Lachnospira* abundance presented significant correlations with the number of CD4 + T cells and HIV viral load [100,101], and a significant association between total fiber intake and *Lachnospira* abundance was observed [102]. Lower abundance of *Lachnospira* was observed in HCV-infected patients [103], which could suggest loss of protection and persistent inflammation since this genus is an SCFA-producing taxa. In addition, the lower abundance of *Lachnospira* observed in HCV-infected people was completely abolished after 3 months under sustained viral response, which could suggest increased production of butyrate and, therefore, a healthier gut. Interestingly, *Lachnospira* abundance was decreased in patients with neuroinfection when compared to controls [104].

### 2.7. Lachnospiraceae Unclassified

The family Lachnospiraceae is a morphologically and phylogenetically varied taxon, with a high number of species [105]. One of the main challenges in studying Lachnospiraceae is its diversity, as the family includes numerous members that are difficult to classify at the genus level using 16S rRNA gene sequencing. As a result, many microbiome studies report these bacteria as “Lachnospiraceae unclassified” or “Lachnospiraceae incertae sedis” (unclassified or uncertain placement) [106]. This classification challenge demonstrates the complexity and diversity within this family and highlights the need for more advanced techniques to accurately identify and characterize its members.

In humans, the family Lachnospiraceae has been linked to various diseases. Reduced levels of Lachnospiraceae have been consistently associated with obesity, type 2 diabetes, and metabolic syndrome, and a lower abundance of this family correlates with increased body mass index and insulin resistance, suggesting a role in metabolic regulation [96,107]. High abundance of Lachnospiraceae in the human gut microbiome is related to protection against different types of cancer [108,109,110]. In addition, Lachnospiraceae abundance is inversely correlated with cardiovascular disease risk factors [111,112] and there is emerging evidence that Lachnospiraceae may influence the development of allergic diseases [113,114]. As such, a balanced gut microbiota, including this family, may play an important role in immune system development and the prevention of allergies.

In COVID-19, several studies have reported changes in Lachnospiraceae populations. Albrich et al. (2022) demonstrated that Lachnospiraceae was associated with protective immune responses and its abundance was reduced in high-risk COVID-19 groups [74]. Maeda et al. (2020) reported that the abundance of Lachnospiraceae was lower in severe COVID-19 cases compared to healthy controls [59], while Galperine et al. (2023) identified a reduction in the abundance of members of Lachnospiraceae in ventilated COVID-19 patients compared to non-ventilated COVID-19 patients [48]. Schult et al. (2022) also observed a reduction in Lachnospiraceae abundance in severe and fatal COVID-19 cases [61]. Zuo et al. (2020a) identified higher abundance of Lachnospiraceae in stool samples with low or no infectivity of SARS-CoV-2, as well as other SCFA-producing bacteria, while fecal samples with high infectivity exhibited a higher abundance of opportunistic bacteria [115]. These findings suggest that Lachnospiraceae levels may be disrupted in COVID-19, potentially affecting disease severity and progression, and that higher levels may be associated with an increased ability to combat SARS-CoV-2 infection in the gut.

The involvement of Lachnospiraceae in other viral infections has been mainly studied in HIV. San-Juan-Vergara et al. (2018) reported, for the first time, a predominantly Lachnospiraceae-based signature in HIV-infected individuals [116]. Vujkovic-Cvijin et al. (2020) showed HIV-associated dysbiosis characterized by depletion of Lachnospiraceae, regardless of sexual preference [117]. Interestingly, although several previous studies have reported members of the Lachnospiraceae family associated with healthy individuals, the abundance of Lachnospiraceae UCG-004 was increased in a cohort of 13 HIV-positive patients [65,118]. In patients with H1N1, Lachnospiraceae populations were drastically reduced compared to healthy controls, and the reduction was negatively correlated with inflammatory cytokine levels in mouse models of COPD co-infected with H1N1 and *Haemophilus influenzae,* demonstrating its anti-inflammatory potential in viral infections [5,119].

### 2.8. Oscillospira

*Oscillospira* is a genus of anaerobic, Gram-positive bacteria within the family Ruminococcaceae. As SCFA-producers, this genus is important for maintaining gut homeostasis, and lower levels of *Oscillospira* are associated with obesity and metabolic syndrome, suggesting its role in regulating energy metabolism and gut health [120,121]. In addition, *Oscillospira* abundance was inversely correlated with Crohn’s disease [122], nonalcoholic fatty liver diseases [123], and gastric cancer [124].

In COVID-19, alterations in the abundance of *Oscillospira* have been documented in a few studies. Gaibani et al. (2021) identified a correlation between elevated levels of *Oscillospira* and non-ICU COVID-19 patients, suggesting that higher concentrations of this genus may be associated with milder disease manifestations [23]. On the other hand, Schult et al. (2022) reported a significant reduction in *Oscillospira* abundance in severe and fatal COVID-19 cases, indicating that lower levels of these beneficial bacteria may correlate with adverse clinical outcomes [61]. These findings indicate that the abundance of *Oscillospira* may be disrupted in COVID-19, with the observed association between increased *Oscillospira* abundance and less severe cases, alongside the decline noted in severe cases, suggesting a potential role for these bacteria in modulating the inflammatory response and influencing disease progression.

The contribution of *Oscillospira* in the context of other viral infections has presented mixed results. Wang et al. (2020) observed enrichment of this genus in HIV-infected individuals [125], while Chandiwana et al. (2023) observed a low abundance [126]. In elite controllers of HIV, *Oscillospira* abundance showed a significant positive correlation with the CD4/CD8 ratio among HIV-positive individuals, indicating an association with reduced systemic inflammation within this cohort [64]. Furthermore, *Oscillospira* was found to be abundant in these individuals, and their microbiota profile closely resembled that of HIV-negative controls.

### 2.9. Roseburia

*Roseburia* is an SCFA-producing genus of Lachnospiraceae and important for gut health and metabolic regulation, thereby playing a protective role against gastrointestinal disorders and conditions like IBD and ulcerative colitis [127,128,129]. Higher levels of *Roseburia* are associated with improved metabolic health, including lower body weight and enhanced insulin sensitivity, which may help to prevent obesity and type 2 diabetes [130,131]. Furthermore, *Roseburia* may influence cardiovascular health by improving lipid profiles and reducing systemic inflammation [132] and reducing the risk of acute myocardial infarction [133], as well as having potential effects on mental health through the gut-brain axis [134].

In COVID-19, several studies have consistently reported decreased levels of *Roseburia* in patients. Albrich et al. (2022) [74], Tao et al. (2020) [135], Gu et al. (2020) [5], Moreira-Rosário et al. (2020) [99], Schult et al. (2022) [61], and Reinold et al. (2021) [45] observed lower *Roseburia* abundance in severe or high-risk COVID-19 cases. Fan et al. (2023) found reduced *Roseburia* abundance in severe compared to moderate cases [136], while Galperine et al. (2023) reported lower levels in ventilated versus non-ventilated patients [48]. These data suggest that lower levels of this beneficial bacterium may be associated with worse disease outcomes.

A few studies have observed changes in *Roseburia* abundance in other viral infections. In HIV, dysbiosis and reduced levels of Roseburia were linked to increased systemic inflammation and immune activation [56]. In addition, butyric acid levels were positively associated with *Roseburia* abundance [137]. In H9N7 patients, *Roseburia* abundance decreased drastically in intestinal samples compared to healthy controls [93]. Interestingly, *Roseburia* abundance was decreased in patients with neuroinfection when compared to controls [104].

### 2.10. Ruminococcus

*Ruminococcus*, as an SCFA-producing genus of Lachnospiraceae, plays an important role in human health through its ability to ferment complex carbohydrates, produce beneficial SCFAs, maintain gut barrier integrity, and modulate immune responses. Its presence is associated with metabolic health and reduced inflammation, making it an important component of a healthy gut microbiome [138,139]. Changes in *Ruminococcus* populations have been observed in several diseases, including Crohn’s disease [140], IBD [141] and major depression [142], while its increased relative abundance is associated with reduced cardiovascular risk [143].

In COVID-19, changes in *Ruminococcus* populations were observed in several studies. Albrich et al. (2022) found that *Ruminococcus* levels were lower in high-risk COVID-19 groups compared to healthy controls [74]. Similarly, Schult et al. (2022) observed a reduction in *Ruminococcus* abundance in severe and fatal COVID-19 cases [61], suggesting that lower levels of these beneficial bacteria may be associated with worse disease outcomes. Gaibani et al. (2021) noted enrichment of *Ruminococcus* populations in COVID-19 patients who had not been admitted to the ICU [23], indicating that higher levels of this genus may be associated with less severe disease. Zuo et al. (2020b) reported that COVID-19 patients treated with antibiotics showed a reduction in *Ruminococcus obeum* abundance compared to patients not treated with antibiotics [6]. Zhang et al. (2023a) identified that *Ruminococcus* abundance was also reduced in post-COVID-19 patients, depleted in symptomatic recovered patients, and was negatively related to C-reactive protein and natural killer cells [75], suggesting that the reduction in *Ruminococcus* abundance may also persist in the post-recovery phase, potentially aiding in the recovery process. This decrease in abundance across various stages of the disease highlights the potential importance of *Ruminococcus* in modulating the immune response and overall gut health during and after COVID-19, as well as the consequences of antibiotic treatments.

In COVID-19, changes in *Ruminococcus* populations have been observed across several studies. Albrich et al. (2022) [74] and Schult et al. (2022) [61] found lower levels of *Ruminococcus* in high-risk and severe COVID-19 cases, suggesting an association with worse outcomes. On the other hand, Gaibani et al. (2021) reported higher *Ruminococcus* levels in patients who avoided ICU admission [23], indicating a potential link to less severe disease. Zuo et al. (2020b) noted reduced *Ruminococcus obeum* abundance in antibiotic-treated patients [6], while Zhang et al. (2023a) found decreased *Ruminococcus* abundance in post-COVID-19 patients [75], associated with lower C-reactive protein and natural killer cells. These findings suggest that *Ruminococcus* may play a role in immune response and gut health during and after COVID-19, with antibiotic treatment potentially impacting its levels.

In other viral infections, significant changes in *Ruminococcus* abundance were also observed in different studies. Gu et al. (2020) demonstrated that the reduction in *Ruminococcus* populations is a biomarker capable of differentiating COVID-19 patients from H1N1 patients [5]. In H7N9 patients, *Ruminococcus* abundance was reduced in intestinal samples compared to healthy controls [93]. In patients with cirrhotic hepatitis C, *Ruminococcus* abundance is also characteristically reduced [144]. Members of this genus express Histo-blood group antigens (HBGA)-like molecules, which are putative receptors for viruses. In stool samples from children with rotavirus, a binding interaction between *Ruminococcus* and rotavirus was identified and confirmed in vitro [145]. The presence of *Ruminococcus* was also associated with lower IgA titers against rotavirus in adults, which demonstrates how this taxon can negatively affect rotavirus infection (Rodríguez-Díaz et al., 2017) [146]. In HIV, dysbiosis and reduced levels of *Ruminococcus* have been linked to increased systemic inflammation and immune activation [56].

## 3. Probiotic Supplementation as Adjunctive Treatment for COVID-19

Probiotic supplementation has been widely studied for its potential health benefits, particularly in maintaining gut health, modulating immune responses, and preventing or alleviating various diseases. Consequently, probiotics and microbiota-targeted therapies hold significant potential as adjunctive treatments for COVID-19. By modulating immune responses, enhancing gut barrier integrity, and preventing secondary infections, these therapies could improve clinical outcomes and reduce disease severity. Most studies of COVID-19 with probiotic supplementation have used *Bifidobacterium* or *Lactobacillus* (or a combination of both), as explored in our previous reviews focusing on these two genera [16,17,18]. While these probiotics have shown promise in modulating immune responses and improving gut health, there is a need to expand our current knowledge to include other beneficial genera explored in this study to fully understand their potential in managing COVID-19. Here, we summarize the potential mechanisms and benefits of using these genera as adjunctive treatment for COVID-19.

### 3.1. Akkermansia Supplementation

*Akkermansia* supplementation can modulate the immune system by promoting the production of anti-inflammatory cytokines and reducing pro-inflammatory responses [21]. This immune modulation could help to alleviate the hyperinflammatory response observed in severe COVID-19 cases. In addition, pasteurized *Akkermansia muciniphila* supplementation improves insulin sensitivity and reduces blood marker levels of liver dysfunction and inflammation [147]. Furthermore, oral administration of *Akkermansia* has also been shown to enhance mouse defenses against the H7N9 virus, significantly reducing viral loads of IL-6 and IL-1β in the lungs and levels of IL-6 and TNF-α in the blood during H7N9 infection. Therefore, suppressing these cytokines should improve the outcomes of the viral infection [148]. A membrane protein in *Akkermansia*, Amuc_1100, interacts with Toll-like receptor 2 (TLR) to bolster intestinal barrier function and can activate TLR4, increasing IL-10 production [22,149]. Currently, the effects of pasteurized *Akkermansia muciniphila* in reducing symptoms related to irritable bowel syndrome, as well as its potential benefits for anxiety and depression, along with its safety and tolerability, are being evaluated in clinical trials (NCT05348642). These studies could establish *Akkermansia muciniphila* as a viable therapeutic option for managing gut health, improving host antiviral immune responses, and addressing associated mental health conditions like anxiety and depression.

In addition, live *Akkermansia muciniphila* has been shown to protect against lethal sepsis in a murine model. This species can produce a tripeptide Arg-Lys-His (RKH), which also protects against organ damage associated with sepsis, reduces pulmonary inflammation, and decreases the overproduction of pro-inflammatory factors [150]. This protective ability against sepsis is particularly important in the context of COVID-19, as SARS-CoV-2 can induce sepsis independent of secondary bacterial or fungal infections [151].

*Akkermansia muciniphila* can also protect against *Clostridioides difficile* infection in mice, where its oral administration prevented weight loss; reduced histological colon damage; significantly lowered levels of G-CSF, MCP-1, IL-17A, IL-1α, IL-6, and TNF-α; and increased the production of SCFAs [152]. In vitro, both live and dead bacteria, as well as their supernatant, alleviated inflammation, improved intestinal barrier function, and modulated the production of IL-1β, TNF-α, and IL-10 [153]. The ability to prevent infections, like secondary infections, is essential in the context of COVID-19, as it can help to avoid additional complications and the deterioration of the patient’s health.

### 3.2. Bacteroides Supplementation

While direct studies on *Bacteroides* supplementation in COVID-19 are limited, the existing research supports further exploration of its potential benefits. In mouse models infected with Influenza A, administration of *Bacteroides dorei* led to increased survival, improved lung pathology, and reduced weight loss, lung index, and colon length [154]. It also reduced the viral load in lung tissue and increased the expression of type 1 interferon at 3 days post-infection. At 7 days post-infection, it decreased lung and serum levels of interferon, IL-1β, IL-6, TNF-α, IL-10, MCP-1, and IP-10. *Bacteroides dorei* treatment also altered the gut microbiota, which may contribute to the restoration of a healthy microbiota and its beneficial effects. *Bacteroides thetaiotaomicron*, a next-generation probiotic, is currently being developed as a biotherapeutic for Crohn’s disease. It has demonstrated good tolerability and safety in clinical trials, including the first human safety study, which is relevant for expanding its potential applications (NCT02704728) [155]. These bacteria have the ability to digest dietary fibers and host glycans while producing short-chain and organic acids, and their cell-surface architecture facilitates both interactions with and evasion of the host immune system [156]. These features make *Bacteroides* a promising candidate for supplementation, particularly in modulating the gut microbiome and enhancing immune responses, which are important for the treatment of inflammatory and infectious diseases, such as COVID-19.

### 3.3. Blautia Supplementation

*Blautia* supplementation may also modulate the immune system, promoting anti-inflammatory production and lessening pro-inflammatory responses, which could help to address hyperinflammatory reactions in severe COVID-19 cases [63]. Increasing *Blautia* abundance through dietary interventions, such as high-fiber diets, has been associated with improved gut health and reduced inflammation [63]. The efficacy and safety of Blautix, a strain of *Blautia hydrogenotrophica*, has been tested for irritable bowel syndrome, showing improvements in intestinal pain and bowel habits [157]. It was demonstrated in a mouse model that the administration of *Blautia faecis* protected against secondary bacterial infections caused by *Streptococcus pneumoniae*. Mice infected with Influenza A and treated with *Blautia faecis* showed reduced bacterial load in the lungs, lower bacterial counts in the spleen, indicating less bacterial dissemination from the lungs, and improved survival. This was associated with less post-influenza weight loss and faster weight recovery. Additionally, *Blautia* administration also reduced the expression of genes encoding inflammatory cytokines, such as TNFα, IL-1β, OAS3, and ISG15 [65]. These findings highlight the potential of *Blautia*, which plays an important role in modulating the inflammatory response and protecting against secondary bacterial infections, as observed in COVID-19.

### 3.4. Coprococcus Supplementation

In mouse models of colitis, the administration of *Coprococcus eutactus* improved colitis by significantly reducing levels of pro-inflammatory cytokines TNF-α, IL-1β, and IL-6, while increasing those of IL-10, IL-4, and IL-5 [158]. It also restored tight junction integrity and epithelial barrier function by elevating levels of key proteins and mucin genes *Coprococcus* can reduce inflammation and improve epithelial barrier integrity, both of which are important for controlling COVID-19, as these clinical features are often disrupted in the disease. Additionally, *Coprococcus* has been linked to improved mental health outcomes by producing metabolites that influence the gut–brain axis [159]. In this context, patients with irritable bowel syndrome exhibit microbiota profiles like those with depression, both showing a decrease in *Coprococcus* abundance [160]. This relationship suggests that intestinal inflammation may interfere with communication with the central nervous system [161], which is particularly relevant for COVID-19 patients, who often experience neurological and psychological symptoms. Future clinical trials should evaluate *Coprococcus*-based probiotics in COVID-19 treatment, focusing on disease severity, recovery time, and overall health, including potential benefits for neurological and psychological symptoms.

### 3.5. Faecalibacterium Supplementation

Several studies have shown that *Faecalibacterium prausnitzii* supplementation can reduce inflammation and improve gut barrier function in animal models of colitis, with ongoing clinical trials evaluating its efficacy in human IBD patients [162]. In mice, *Faecalibacterium duncaniae* provided protection against influenza infection, restoring SCFA levels in infected animals, impairing viral replication in the lungs, and reducing pulmonary inflammation, in addition to demonstrating potential protection against secondary bacterial infections [163]. In chronic kidney disease models, *Faecalibacterium prausnitzii* improved intestinal permeability and inflammation, primarily through the beneficial effects of butyrate produced by *Faecalibacterium prausnitzii* [164]. To increase the presence of *Faecalibacterium prausnitzii* in Crohn’s disease, a study evaluated the use of fructooligosaccharides to increase the abundance of this taxon (NCT02539849). Another clinical trial is assessing the EXL01 strain of *Faecalibacterium prausnitzii* for maintaining clinical response/remission induced by corticosteroids and preventing recurrence of *Clostridioides difficile* infection in high-risk patients, as a low abundance of *Faecalibacterium* prausnitzii is predictive of *Clostridioides difficile* recurrence (NCT06306014), with completion expected by 2027.

These studies indicate that supplementation with *Faecalibacterium* or strategies to increase its abundance may help reduce intestinal inflammation, improve gut barrier integrity, and regulate the immune system, as well as prevent recurrences of infections such as those caused by *Clostridioides difficile*, which may be particularly relevant in the context of COVID-19.

### 3.6. Lachnospira and Lachnospiraceae Supplementation

*Lachnospira* and Lachnospiraceae species produce SCFAs and may help manage hyperinflammatory responses in severe COVID-19 cases. Furthermore, *Lachnospira* and Lachnospiraceae are linked to reduced inflammation and improved gut health, making them potential therapeutic targets for COVID-19 patients with comorbidities like IBD and metabolic disorders [165,166]. Increasing the abundance of these bacteria through high-fiber diets has been associated with improved gut health and reduced inflammation [167]. Furthermore, isolates of Lachnospiraceae can significantly reduced colonization by *Clostridioides difficile* in mouse models and lowered levels of *Clostridioides difficile* cytotoxins, resulting in less severe clinical and histopathological signs and reduced mortality [168]. These findings suggest that *Lachnospira* and Lachnospiraceae contribute to gut health and inflammation reduction and have significant therapeutic potential for the prevention and management of secondary bacterial infections, as seen in COVID-19.

### 3.7. Oscillospira Supplementation

Different studies have shown that *Oscillospira* can produce a variety of SCFAs, especially butyrate [120,169,170], which indicates that the potential immune modulation by these bacteria may help to alleviate hyperinflammatory responses in severe COVID-19 cases. In addition, *Oscillospira* is associated with leanness and overall metabolic health [171], making it a potential therapeutic target for COVID-19 patients with comorbidities like obesity and metabolic syndrome [172]. By potentially improving gut health and enhancing the immune response, *Oscillospira* supplementation could serve as a therapeutic target for these vulnerable populations. Its ability to modulate inflammation and promote metabolic health may help to reduce the risks associated with COVID-19 in patients suffering from these comorbidities [172]. As studies exploring new probiotics continue to unfold, targeting *Oscillospira* may provide a novel approach to improving outcomes for individuals with underlying health issues during viral infections.

### 3.8. Roseburia Supplementation

By producing butyrate, *Roseburia* enhances gut barrier integrity, preventing the translocation of pathogens and toxins into the bloodstream, a key function given the gut barrier dysfunction often observed in COVID-19 patients [7]. The administration of a butyrate-producing strain of *Roseburia hominis* in rat models prevented the development of visceral hypersensitivity. In addition, supplementation prevented the reduction in mRNA expression of occludin protein, which is essential for the formation of tight junctions, and increased butyrate levels [173]. Supplementation with *Roseburia intestinalis* in murine models has also been shown to improve impaired colon barrier function, restore gastrointestinal function, and recover the microbiota, as well as reduce serum levels of IL-6 and IL-17 [129]. Furthermore, *Roseburia intestinalis* has the ability to reverse memory impairment [174], which is an important approach, especially for post-COVID-19 patients [175].

*Roseburia* abundance is also inversely correlated with the development of atherosclerotic lesions [132]. In this context, COVID-19 and atherosclerosis exhibit a bidirectional relationship, where cardiovascular diseases increase susceptibility to viral infections, and SARS-CoV-2 infection can damage the endothelium and cardiomyocytes, exacerbating atherosclerosis [176,177]. Targeting the gut microbiota to enhance *Roseburia* levels may offer a novel therapeutic approach that could improve outcomes for individuals with underlying heart conditions during viral infections. This strategy could promote gut health, modulate the immune system, and even reverse memory impairment, making it a promising approach for at-risk groups, acute diseases, and post-COVID-19 recovery.

### 3.9. Ruminococcus Supplementation

*Ruminococcus* species degrade complex polysaccharides and produce SCFAs, including butyrate [178]. *Ruminococcus bromii*, for example, is specialized in breaking down resistant starch [179], which is prevalent in foods such as cereal grains, beans, lentils, and certain fruits like plantains and green bananas. Through this process, *Ruminococcus bromii* produces SCFAs, including acetate, propionate, and butyrate, which are vital for gut health. Similarly, bifidobacteria are recognized for their substantial repertoire of starch-degrading enzymes, enabling them to effectively utilize resistant starch as well [180]. As such, the combination of strains of these two genera could significantly enhance starch degradation and SCFA production, particularly in the context of COVID-19. Notably, *Ruminococcus bromii* and *Bifidobacterium adolescentis* are known as primary degraders of resistant starch in the human gut [179]. Therefore, the combination of *Ruminococcus bromii* and *Bifidobacterium adolescentis* could not only enhance resistant starch degradation but also contribute to a more balanced immune response and reduced inflammation, helping to alleviate the adverse effects of the virus on the body. This integrated approach could open new avenues for dietary and probiotic interventions in patients affected by COVID-19.

In mouse models of induced colitis, the administration of *Ruminococcus intestinalis* significantly reduced IL-17 expression and improved pathological signs of inflammation in the colon compared to mice that did not receive the treatment [181]. Furthermore, in hamsters infected with SARS-CoV-2, administration of a *Ruminococcus* strain resulted in no clinical symptoms of SARS-CoV-2 infection, improved survival rates and body weight, and no signs of pneumonia were observed. Gross examination of the lung specimens demonstrated protective effects against the infection [182]. Additionally, *Ruminococcus torques* is being evaluated in healthy overweight individuals for its potential metabolic effects, including metabolism, energy, glucose regulation, and inflammatory response (NCT05448274). These studies suggest that different species of *Ruminococcus* may have protective and therapeutic properties in various conditions, such as intestinal inflammation, with the potential to modulate the immune system, improve metabolic health, and even address viral infections like COVID-19.

### 3.10. Key Points on Probiotics Supplementation and COVID-19 Treatment

The studies presented in this section indicate that various probiotic bacterial genera may significantly improve clinical outcomes and decrease the severity of COVID-19. These beneficial bacteria could positively influence patient health by reducing disease severity and speeding up recovery. Table 1 summarizes the mechanisms of action, health benefits, clinical evidence, and ongoing research for the different probiotic genera that have shown promise in the context of COVID-19 treatment. However, to confirm these advantages, future clinical trials are necessary to assess the effectiveness of probiotics derived from these species in treating COVID-19. Studies should concentrate on key factors like disease severity, recovery time, and overall patient well-being. Conducting these trials is of primary relevance to determining whether probiotic administration could serve as an effective strategy for managing COVID-19, particularly regarding clinical management and the quality of life for those affected. Ongoing investigations into the role of these microorganisms in influencing immune responses and supporting gut health show promise, potentially leading to new complementary treatments that alleviate the virus’s negative effects. Therefore, continuous exploration of the relationship between gut microbiota and COVID-19 is essential for developing new efficient therapeutic approaches.

## 4. Concluding Remarks

This review has explored the relationship between nine key genera (*Akkermansia*, *Bacteroides*, *Blautia*, *Coprococcus*, *Faecalibacterium*, *Lachnospira*, *Oscillospira, Roseburia*, and *Ruminococcus*), as well as the family Lachnospiraceae, and their relevance to COVID-19. Each of these genera plays a major role in maintaining gut homeostasis, producing SCFAs, and modulating systemic inflammation. Alterations in their abundance have been observed in COVID-19 patients, with trends suggesting that disruptions in populations of these beneficial bacteria are associated with worse disease outcomes. Furthermore, the relationship between these genera and other viral infections, such as HIV and influenza, suggests that maintaining a healthy gut microbiota could be beneficial in mitigating the effects of viral diseases. The production of SCFAs like butyrate by these bacteria enhances gut barrier integrity and reduces systemic inflammation, which are critical during viral infections. Importantly, emerging evidence suggests that the dysbiosis observed in COVID-19 may also play a role in the development of long COVID, a condition characterized by prolonged symptoms following acute infection. Understanding how these gut microbiota alterations contribute to long COVID could provide valuable insights for therapeutic interventions. In summary, maintaining a healthy microbiota, particularly the beneficial genera discussed in this review, is essential for immune function and overall health. As we continue to combat the COVID-19 pandemic, integrating microbiota-focused approaches into treatment strategies could provide new opportunities for improving patient outcomes during acute infection but also offer potential pathways for managing long COVID and enhancing recovery.

## Figures and Tables

**Table 1:** Comparison of probiotic genera discussed in this study for adjunctive treatment of COVID-19.

Genus	Mechanisms of Action	Health Benefits	Clinical Evidence	Ongoing Research/Trials
*Akkermansia*	Modulates immune response, enhances gut barrier integrity, produces anti-inflammatory cytokines	Improves insulin sensitivity, reduces liver dysfunction, protects against viral infections	Reduces IL-6 and TNF-α levels in H7N9 virus models; protective in sepsis models	Trials on irritable bowel syndrome and mental health conditions (NCT05348642)
*Bacteroides*	Alters gut microbiota, digests dietary fibers, produces SCFAs	Increases survival in Influenza A models, improves gut health	Increases type 1 interferon expression; reduces inflammatory cytokine levels in mouse models	Development as a biotherapeutic for Crohn’s disease (NCT02704728)
*Blautia*	Promotes anti-inflammatory cytokine production, modulates gut health	Protects against secondary bacterial infections, improves bowel habits	Reduces bacterial load in Influenza A models; improves survival	Efficacy of Blautix for irritable bowel syndrome [157]
*Coprococcus*	Reduces pro-inflammatory cytokine levels, restores epithelial barrier function	Improves mental health outcomes, influences gut-brain axis	Linked to reduced inflammation in colitis models; associated with mental health	Future trials to evaluate neurological benefits in COVID-19 patients
*Faecalibacterium*	Improves gut barrier function, produces butyrate	Reduces intestinal inflammation, protects against infections	Provides protection against influenza; improves gut health in chronic kidney disease models	Trials on maintaining clinical response in Crohn’s disease (NCT02539849)
*Lachnospira*	Produces SCFAs, reduces inflammation	Improves gut health, may help manage hyperinflammatory responses	Reduces Clostridioides difficile colonization in mouse models	Potential therapeutic targets for COVID-19 patients with comorbidities
*Oscillospira*	Produces SCFAs, associated with metabolic health	Alleviates hyperinflammatory responses, supports overall gut health	Linked to metabolic health; potential impact on obesity-related COVID-19 risks	Targeting *Oscillospira* for therapeutic approaches in viral infections
*Roseburia*	Produces butyrate, enhances gut barrier integrity	Prevents pathogen translocation, improves memory function	Improves colon barrier function and reduces inflammatory cytokines in murine models	Investigating role in cardiovascular health and COVID-19 recovery
*Ruminococcus*	Degrades polysaccharides, produces SCFAs	Modulates immune response, improves metabolic health	Protects against SARS-CoV-2 in hamster models; reduces inflammation in colitis models	Evaluating metabolic effects in overweight individuals (NCT05448274)

## Data Availability

No new data were created or analyzed in this study.
